# Visual liver assessment using Gd-EOB-DTPA–enhanced magnetic resonance imaging of patients in the early post-Fontan period

**DOI:** 10.1038/s41598-020-61618-7

**Published:** 2020-03-17

**Authors:** Kimiko Nakajima, Mitsuru Seki, Shinitsu Hatakeyama, Shuhei Arai, Yuji Asami, Kensuke Tanaka, Kentaro Ikeda, Shinya Shimoyama, Tomio Kobayashi, Takashi Miyamoto, Yasunori Okada, Hirokazu Arakawa, Takumi Takizawa

**Affiliations:** 1 Department of Cardiology, Gunma Children’s Medical Center, Gunma, Japan; 2 Department of Pediatrics, Gunma University Graduate School of Medicine, Gunma, Japan; 3 Department of Radiology, Gunma Children’s Medical Center, Gunma, Japan; 4 Department of Cardiovascular Surgery, Gunma Children’s Medical Center, Gunma, Japan

**Keywords:** Diagnostic markers, Congenital heart defects

## Abstract

No imaging modality can be used to evaluate Fontan-associated liver disease (FALD). We retrospectively reviewed hepatic gadolinium ethoxybenzyl diethylenetriamine pentaacetic acid-enhanced magnetic resonance imaging (EOB-MRI) characteristics of patients within 1 year post-Fontan procedure, and we evaluated the association between hepatic imaging abnormalities and clinical parameters, including follow-up cardiac catheterization and laboratory test findings. The EOB-MR images were graded, based on the extent of the decreased enhancement, as “normal” (Grade 1), “segmental” (Grade 2), “regional” (Grade 3), and “diffuse” (Grade 4). We enrolled 37 patients (mean age, 3.5 ± 1.0 years): 9 patients had Grade 1 or 2; 14 patients, Grade 3; and 14 patients, Grade 4. EOB-MRI revealed characteristic reticular or mosaic patterns of diminished enhancement (i.e. “frog spawn” appearance). Ultrasonography did not detect diminished enhancement or “frog spawn” appearance. A trend existed toward increased grade severity in imaging with increased central venous pressure, pulmonary vascular resistance, and gamma-glutamyltransferase levels. Noninvasive EOB-MRI revealed the characteristic pattern of diminished enhancement, which was correlated with certain clinical parameters indicative of Fontan physiology and liver dysfunction. Early-stage FALD may occur soon after the Fontan procedure and is associated with increased pressure in the inferior vena cava and hepatic veins.

## Introduction

The Fontan procedure, a palliative surgical procedure used in patients with a single-ventricle heart, has gained widespread recognition since its introduction in 1968^1^. It is widely adapted to various forms of functional single-ventricle physiology. Recent advances in surgical and postoperative management have enabled most Fontan patients to survive far into adulthood^2^. Therefore, the diagnosis and treatment of long-term complications of the procedure have become increasingly important.

The Fontan circulation separates venous return from the heart, which allows volume unloading of a single ventricle and permits normal arterial oxygen saturations; however, the Fontan circulation elevates central venous pressure and decreases cardiac output^3,4^. A critical long-term noncardiac complication of the Fontan procedure is Fontan-associated liver disease (FALD)^5–7^. FALD manifests as hepatic fibrosis, cirrhosis, ascites, synthetic dysfunction, hepatocellular carcinoma (HCC), and portal hypertension. Long-term studies^8,9^ of patients post-Fontan procedure demonstrate with liver biopsy that hepatic fibrosis progresses throughout follow up. Liver biopsy is the gold standard for evaluating liver fibrosis. However, it is invasive and not routinely performed in young Fontan patients because of anaesthetic issues and possible complications, especially in patients who undergo anticoagulant treatment. In addition, liver biopsy only addresses limited lesions and evaluating the entire liver is difficult. These factors have constrained scientists’ understanding of the aetiology, frequency, and time frame for the development of hepatic dysfunction in young Fontan patients. A noninvasive procedure to assess liver histology needs to be developed.

Gadolinium ethoxybenzyl diethylenetriaminepentaacetic acid (Gd-EOB-DTPA), a hepatobiliary-specific magnetic resonance imaging (MRI) contrast agent, initially behaves as an extracellular contrast agent. After it enters hepatocytes, it is excreted into the biliary system^10^. Therefore, decreased or late enhancement of the liver on MRI with Gd-EOB-DTPA (EOB-MRI) is associated with hepatic dysfunction or failure^11,12^. This noninvasive technique has been established with excellent results in other chronic liver diseases such as HCC, metastatic liver tumours, and cirrhosis^13–15^. However, no study to date has investigated liver abnormalities in Fontan patients. The aims of this study were to describe the imaging characteristics of the liver in hepatic EOB-MRI and to clarify the relationship between hepatic imaging abnormalities and postoperative clinical parameters in young patients soon after the Fontan procedure.

## Results

### Patients’ characteristics

The baseline characteristics of the patients are summarized in Table 1. We investigated 18 male and 19 female patients. The mean age at the MRI evaluation was 3.5 ± 1.0 years (age range, 2–7 years). The mean time interval between the Fontan procedure and the MRI evaluation was 1.2 ± 0.4 years. In this population, 27% of patients had heterotaxy syndrome and 65% had left dominant ventricular morphology. Only one patient underwent the Fontan procedure with an intracardiac conduit, whereas 22 patients underwent the fenestrated Fontan procedure. All patients were administered anticoagulant therapy, including warfarin. No patient exhibited hepatosplenomegaly, oesophageal varix, or active hepatitis infections.

**Table 1:** Patients’ characteristics (*n* = 37).

Total, n	37
Sex, male, *n* (%)	18 (49)
Age at Fontan operation (y), mean ± SD	2.4 ± 0.8
Age at MRI evaluation (y), mean ± SD	3.5 ± 1.0
**Fontan procedure, n (%)**	
Extra-cardiac conduit	36 (97)
Fenestration	22 (59)
**Heterotaxy syndrome, n (%)**	
Right isomerism	6 (16)
Left isomerism	4 (11)
Anatomy, *n* (%)	
**Left ventricle dominant**	
Pulmonary atresia with intact ventricular septum	4 (11)
Tricuspid atresia	4 (11)
Double outlet right ventricle	4 (11)
Single left ventricle	9 (24)
Others	3 (8)
**Right ventricle dominant**	
Unbalanced atrioventricular septal defect	2 (5)
Hypoplastic left heart syndrome	1 (3)
Double outlet right ventricle	1 (3)
Single right ventricle	9 (24)
**Medication**, ***n*** **(%)**	
Anticoagulants	37 (100)
Vasodilators	33 (89)
Pulmonary vasodilators	31 (84)
Diuretics	12 (32)
β-blockers	7 (19)
Antiarrhythmic agents	3 (8)

MRI, magnetic resonance imaging; SD, standard deviation.

### The EOB-MRI findings

**Figure 1:**
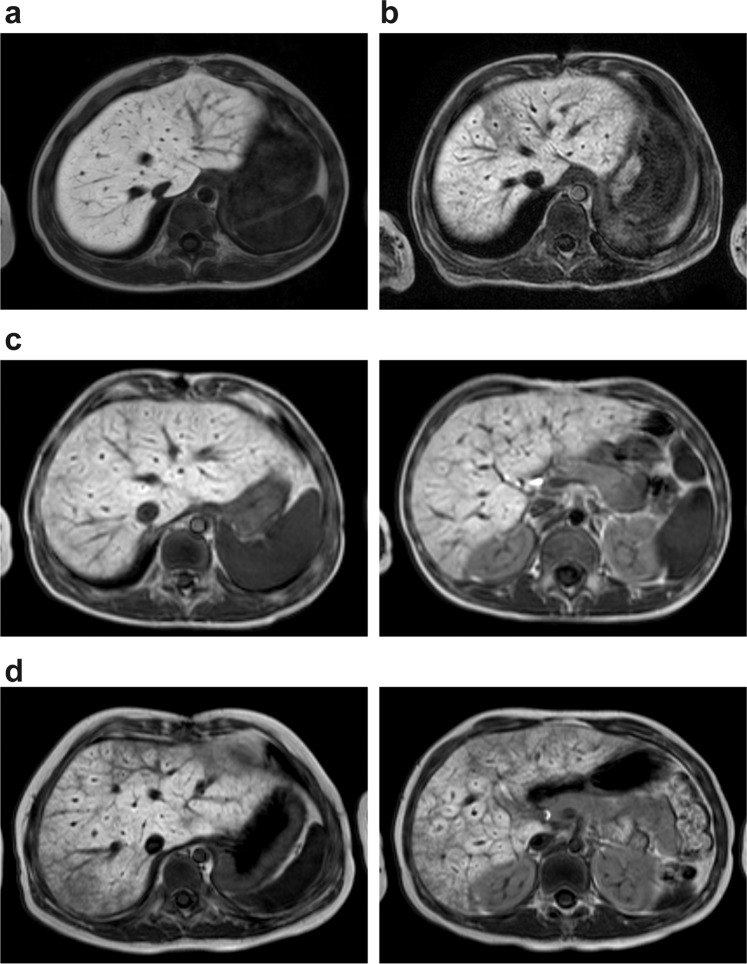
(**a**) Grade 1: normal liver enhancement on gadolinium ethoxybenzyl diethylenetriaminepentaacetic acid magnetic resonance imaging (EOB-MRI). Normal enhancement shows homogeneous enhancement of the liver parenchyma with high signal intensity, a sharp liver periphery, and no dilation of the hepatic veins. (**b**) Grade 2: segmental decreased enhancement of the liver parenchyma around the hepatic vein perfusion area, which indicates mild poor enhancement. (**c**) Grade 3: lobar or diffuse decrease in enhancement on four slices or fewer. Both images are from the same patient. The left panel shows mild diffuse decrease in enhancement, whereas the right panel shows normal enhancement in a different section. (**d**) Grade 4: diffuse decreased enhancement of the liver. Mosaic enhancement of the liver parenchyma involves the hepatic vein perfusion area with a specific enhancement pattern characterised by a heterogeneous “frog spawn-like” enhancement. The panels correspond to different sections from the same patient and show diffuse poor enhancement.

Abnormal enhancement on EOB-MRI was observed in 32 (87%) patients (Table 2). Most of these patients were classified as having decreased contrast enhancement of Grade 3 or Grade 4 (38% each). The typical normal liver findings of homogeneous enhancement of the liver parenchyma with high signal intensity, a sharp liver periphery, and no dilation of hepatic veins (i.e. Grade 1) are depicted in Fig. 1a. Figure 1b illustrates Grade 2, which is a segmental decrease in enhancement around the hepatic vein perfusion area. Figure 1c illustrates Grade 3 in which a lobar or a diffuse decrease in enhancement exists in four slices or fewer. The left image in Fig. 1c depicts a mild decrease in enhancement and the right image depicts normal enhancement. Figure 1d illustrates Grade 4 in which a diffuse decrease in liver enhancement exists on five slices or more. Both panels in Fig. 1d of different sections show diffuse poor enhancement. Decreased enhancement is associated with the characteristic mosaic enhancement of the liver parenchyma. We designated this specific enhancement pattern as the “frog spawn-like” appearance because of its resemblance to frog spawns.

**Table 2:** Summary of EOB-MRI findings.

Findings	*n* (%)
**Range of liver poor enhancement**	
None (Grade 1)	5 (13)
Segmental (Grade 2)	4 (11)
Regional (Grade 3)	14 (38)
Diffuse (Grade 4)	14 (38)
**Hepatic lesions**	
Blunt margins of liver surface	8 (22)
Cholestasis; prolonged excretion of the contrast agent	5 (14)
Hepatic vein dilation	18 (49)
Portal vein dilation	0 (0)
Extrahepatic manifestations of portal hypertension	0 (0)

EOB-MRI, gadolinium ethoxybenzyl diethylenetriaminepentaacetic acid magnetic resonance imaging.

Note: The total number of patients is 37.

**Figure 2:**
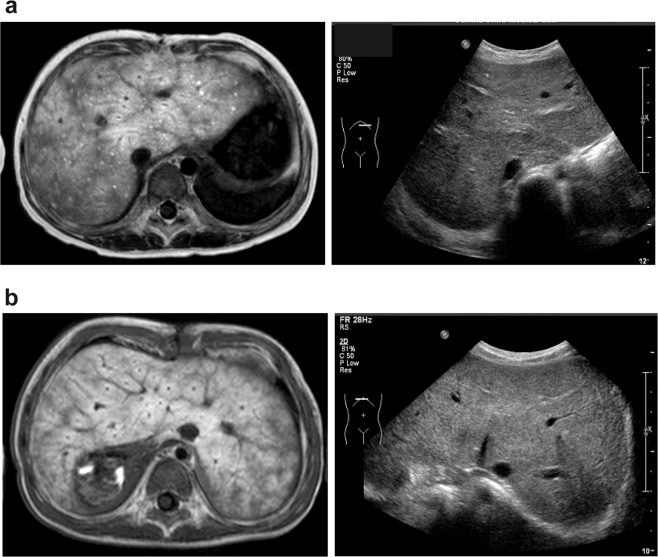
Comparison of gadolinium ethoxybenzyl diethylenetriaminepentaacetic acid (Gd-EOB-DTPA)-enhanced magnetic resonance imaging (i.e. EOB-MRI) and abdominal ultrasonography. (**a**) The patient is a 5-year-old girl with hypoplastic left heart syndrome. The left panel shows the EOB-MRI T1-weighted (T1WI) findings at 1 year after the Fontan procedure. A contrasting defect of the liver parenchyma, inhomogeneous enhancement in the liver periphery, and diffuse high-intensity spots are visible, which indicate cholestasis. In the right panel ultrasonography of the liver shows high echoic spots and a low echoic area. This finding reveals roughening of the liver parenchyma. (**b**) The patient is a 2-year-old boy with right isomerism heart and a single right ventricle. The left panel shows the EOB-MRI (T1WI) findings at 1 year after the Fontan procedure. Reticular contrasting defects of the liver parenchyma that correspond to the hepatic vein area are visible. Its appearance resembles “frog eggs.” The findings of “frog egg” is spread throughout the liver. The surface of liver is rough. The right panel shows the findings of ultrasonography of the liver, which has high echoic spots and a low echoic area.

We next compared transverse slices of the T1-weighted functional EOB magnetic resonance and abdominal ultrasonographic images of the liver at the hypochondrial levels. We found a complete discordance between them. The EOB-MRI images revealed diffuse decreased enhancement in the liver with mosaic enhancement of the parenchyma (Fig. 2a,b, left panels); inhomogeneous enhancement in the blunted margins of the surface; and the portal vein perfusion area with markedly contrasted and diffuse high-intensity spots (Fig. 2a, left panel), which indicated prolonged excretion of contrast agent or cholestasis. In marked contrast, we only found roughening of the liver parenchyma by imaging concordant regions with abdominal ultrasonography that had been performed on the same day (Fig. 2a,b, right panels).

### Comparison of clinical findings with different enhancement grades in EOB-MRI

**Figure 3:**
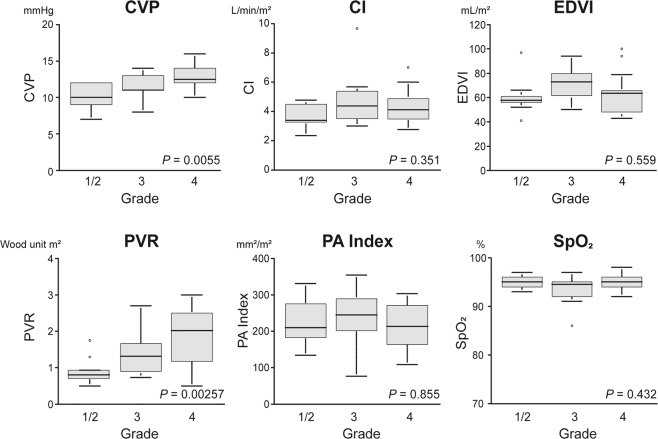
The univariate analysis of hemodynamic findings at catheterization conducted 1 year after the Fontan procedure and the severity grade of liver dysfunction. At the follow-up catheterization conducted 1 year after the Fontan surgery, a trend exists between grade severity and the following measures: central venous pressure (CVP), cardiac index (CI), end-diastolic ventricular volume index (EDVI), pulmonary vascular resistance (PVR), pulmonary artery (PA) index, and saturation of percutaneous oxygen (SpO_2_).

We next compared clinical characteristics, laboratory data at catheterization, and medications between patients with different EOB-MRI grades. The patients’ age at the time of the Fontan procedure was relatively higher for the Grade 4 group, but was not statistically different between the groups (*P* = 0.12) (Table 3). The laboratory data at the time of the cardiac catheterization conducted 1 year after surgery indicated that the serum gamma-glutamyltranspeptidase (GGT) levels were significantly different (*P* = 0.016). The GGT level increased as the grade increased. However, serum levels of aspartate transaminase (AST), alanine aminotransferase (ALT), and alkaline phosphatase were not significantly different between the groups. With regard to parameters measured during the cardiac catheterization, the univariate analysis indicated a trend toward a higher grade with a higher central venous pressure (CVP) (*P* = 0.006) and higher pulmonary vascular resistance (PVR) (*P* = 0.003) (Fig. 3 and Table 4). However, no significant difference in the other hemodynamic parameters existed between the different grades.

**Table 3:** A comparison of the baseline characteristics of the patients, based on the magnetic resonance imaging findings.

EOB-MRI grade	Grades 1/2^a^ (*n* = 9)	Grade 3 (*n* = 14)	Grade 4 (*n* = 14)	*P*
Age at Fontan surgery (y)	2.0 [1.8, 2.4]	2.0 [1.8, 2.7]	2.4 [2.0, 3.3]	0.12
Interval between Fontan surgery and MRI evaluation (y)	1.0 [0.7, 1.2]	1.1 [1.0, 1.5]	1.1 [1.0, 1.4]	0.22
Heterotaxy syndrome, *n* (%)	0 (0)	6 (43%)	4 (29%)	0.20
Total CPB time (min)	410 [392, 432]	474 [446, 642]	477 [367, 718]	0.43
Fenestrated Fontan, *n* (%)	5 (56%)	6 (43%)	11 (79%)	0.19
CPB time (min)	194 [177, 218]	203 [162, 236]	178 [133, 218]	0.29
**Laboratory data at cardiac catheterization**				
Total bilirubin (mg/dL)	0.45 [0.41, 0.77]	0.53 [0.44, 0.88]	0.63 [0.50, 0.75]	0.32
AST (U/L)	39 [37,44]	43 [33,44]	40 [36,46]	0.90
ALT (U/L)	20 [16,22]	19 [14,26]	20 [16,22]	0.52
alkaline phosphatase (U/L)	701 [680, 1001]	945 [670, 995]	871 [724, 1062]	0.79
GGT (U/L)	36 [23,51]	36 [31,51]	68 [34, 152]	0.016
White blood cell (×10^3^/L)	7.4 [6.6, 8.8]	7.6 [7.0, 8.8]	8.9 [7.0, 11.1]	0.34
Platelet (x10^3^/⌠L)	248 [196, 307]	263 [225, 325]	251 [202, 329]	0.73
Hyaluronic acid (ng/mL)	30 [18,35]	30 [19,41]	31 [18,69]	0.43
Type IV collagen (ng/mL)	258 [214, 316]	253 [230, 292]	300 [229, 369]	0.56
Procollagen III peptide (U/mL)	1.6 [1.3, 1.9]	1.5 [1.2, 1.7]	1.4 [1.2, 1.8]	0.84
BNP (pg/mL)	15 [10,19]	13 [6,20]	25 [14,55]	0.17
**Medication**				
Anticoagulant, *n* (%)	9 (100)	14 (100)	12 (100)	NA
Diuretic, *n* (%)	3 (33)	3 (21)	6 (50)	0.36
Pulmonary vasodilator, *n* (%)	9 (100)	10 (71)	12 (85)	0.49
Vasodilator, *n* (%)	8 (89)	12 (86)	13 (86)	0.71
Beta-blocker, *n* (%)	3 (33)	0 (21)	4 (33)	0.82
Antiarrhythmic agents, *n* (%)	0	3	0	0.78

^a^We combined Grade 1 and 2 because Grade 2 showed only minor abnormalities in EOB-MRI.

Note: The data are presented as the median [interquartile range (25^th^–75^th^ percentile)], unless otherwise indicated.

CPB, cardiopulmonary bypass; AST, aspartate transaminase; ALT, alanine aminotransferase; GGT, gamma-glutamyltranspeptidase; BNP, brain natriuretic peptide; EOB-MRI, gadolinium ethoxybenzyl diethylenetriaminepentaacetic acid magnetic resonance imaging; NA, not applicable.

**Table 4:** Comparison of the haemodynamic findings, based on magnetic resonance imaging findings.

EOB-MRI grade	Grades 1/2 (*n* = 9)	Grade 3 (*n* = 14)	Grade 4 (*n* = 14)	*P*
**Haemodynamics**				
CVP (mmHg)	10 [9,12]	11 [11,13]	13[12,14]	0.0055
CI (L/min/m^2^)	3.40 [3.3, 4.5]	4.4 [3.5, 5.3]	4.1 [3.5, 4.8]	0.351
EF (%)	64 [62,65]	60 [53,62]	66 [59,72]	0.293
EDP (mmHg)	7 [6,9]	6 [5,7]	6 [6,8]	0.556
EDVI (mL/m^2^)	57.6 [55.8, 61.0]	73.0 [61.7, 79.6]	63.5 [51.1, 65.5]	0.559
PVR (unit⋅m^2^)	0.8 [0.7, 0.9]	1.3 [1.0, 1.6]	2.0 [1.2, 2.5]	0.0026
PAI (mm^2^/m^2^)	210 [183, 275]	245 [206, 283]	213 [170, 270]	0.855
SpO2 (%)	95 [94,96]	94 [92,96]	95 [94,96]	0.432

Note: The data are presented as the median [interquartile range (25^th^–75^th^ percentile)].

EOB-MRI, gadolinium ethoxybenzyl diethylenetriaminepentaacetic acid magnetic resonance imaging; CVP, central venous pressure; CI, cardiac index; EF, ejection fraction; EDP, end-diastolic pressure; EDVI, end-diastolic volume index; PVR, pulmonary vascular resistance; PAI, pulmonary artery index; SpO2, percutaneous oxygen saturation.

## Discussion

In this study, we aimed to describe the imaging characteristics of liver abnormalities in Fontan patients, based on hepatic EOB-MRI, and to clarify the relationship between hepatic imaging abnormalities and postoperative clinical parameters in young patients soon after undergoing the Fontan procedure. We observed two important imaging features. First, MRI using a liver-specific contrast agent, Gd-EOB-DTPA was able to depict hepatic enhancement abnormalities in young patients soon after the Fontan procedure. This finding suggested that hepatic anatomical changes occur, even in the early postoperative period. Second, the degree of decreased enhancement was associated with an elevation in the systemic venous pressure, PVR, and serum GGT level.

To date, imaging modalities suitable for evaluating liver abnormalities in Fontan patients have not been established^16–19^. Liver biopsy, which is the gold standard for diagnosing liver abnormality, is invasive and poses a risk of complications such as bleeding, especially in Fontan patients. Hence, the development of a noninvasive liver imaging examination of post-Fontan patients is imperative.

After Gd-EOB-DTPA enters hepatocytes through active carriers [e.g. organic anion transporting polypeptide 1B1 (OATP1B1) and 1B3 (OATP1B3)] on the sinusoidal membrane, Gd-EOB-DTPA is excreted into the biliary system by multidrug resistance-associated protein 2 (MDRP2) without any metabolic changes^20,21^. Gadolinium EOB-DTPA is transported by the same transporters as bilirubin or indocyanine green, which reflects the whole liver function^11^. Decreased or late liver enhancement by Gd-EOB-DTPA may be associated with hepatic dysfunction or failure^13^. In addition to decreased Gd-EOB-DTPA enhancement, we found a specific enhancement pattern characterized by a heterogeneous “frog spawn-like” appearance, which results from poor liver parenchymal enhancement, primarily around hepatic veins.

We assume mechanisms that cause the “frog spawn-like” appearance are as follows. Marked elevation in the blood pressure of the inferior vena cava and hepatic veins after the Fontan procedure causes hepatic congestion and stretch insults. These factors result in poor enhancement (i.e. low signal intensity) on EOB-MRI. This poor enhancement primarily occurs around the hepatic veins. The increase in CVP causes blood flow stasis from the portal vein to the central vein, which results in sinusoidal congestion and ischemia. Diminished oxygen delivery in the hepatic vein region caused by lower oxygen pressure induces sinusoidal liver dysfunction^5^. During the postoperative period, chronic venous hypertension may promote liver fibrosis from near the hepatic vein. The underlying mechanisms are presumedly vascular shear stress, increased parenchymal stiffness, ischemia, or cytokines, which may directly promote hepatic myofibroblast activation^22^. However, hepatocytes around Glisson’s sheath are well preserved in their function, until the late stage, because of the higher oxygen partial pressure in the portal veins and hepatic arteries in Gleason’s sheath. The Glisson’s sheath consequently has a higher signal intensity than do the hepatic vein regions. This difference in signal intensity between hepatic vein region and the Glisson’s sheath region may cause the “frog spawn-like” appearance. A similar patchy enhancement pattern was reported with EOB-MRI in a case of Budd–Chiari syndrome, which manifests as hepatic venous outflow obstruction and hence ischemic injury to liver cells, regardless of the cause^23^. The aforementioned report and our observation suggest that the “frog spawn-like” appearance is attributed to hypofunction or dysfunction of hepatocytes that is caused by congestion of the hepatic vein.

Previous reports^8,24^ have demonstrated long-term post-Fontan complications such as centrilobular and sinusoidal hepatic fibrosis, based on histological assessments of the liver. A longer duration of Fontan circulation and elevated CVP have been indicated as risk factors for centrilobular and sinusoidal fibrosis. However, several studies^25–27^ have shown hepatic histological abnormalities soon after the Fontan procedure. In particular, Schwartz *et al*.^26^ found at autopsy examination significant portal fibrosis in 61% of patients and sinusoidal fibrosis in 78% of patients who died within 1 year after the Fontan operation. These changes were associated with the length of hospitalization after pre-Fontan cardiac operation and with the pre-Fontan mean right atrial pressure. Johnson *et al*.^28^ also showed portal fibrosis in 30% of patients and sinusoidal fibrosis in 65% of patients who died less than 35 days after the Fontan operation. These studies suggest that the development of FALD depends on long-term Fontan circulation and multiple factors, and may occur very early after the Fontan operation.

A histological assessment was not conducted in the current study. However, our current findings of decreased enhancement with Gd-EOB-DTPA, which likely indicates liver fibrosis, at 1 year after the Fontan operation were consistent with the findings in these previous studies.

The grade of the regional extent of poor enhancement is associated with an elevation in the systemic venous pressure and serum GGT levels (Table 3 and Fig. 3). Serum GGT levels have been implicated in the severity of chronic heart failure in two-ventricular circulation^29^. This finding can be explained by several mechanisms such as hepatic congestion, local damage to the bile canaliculi caused by increased pressure within the hepatic sinusoid, ischemia, and the release of proinflammatory cytokines. However, the precise mechanisms remain unclear. Congestive liver sinusoids likely compress collapsible structures in the lobe (e.g. bile canaliculi and ductules) when hydrostatic pressure and the size of liver cells are increased. This factor can injure the bile canaliculi. We suspect that elevation of GGT levels in Fontan patients (and probably in patients with chronic heart failure) are attributable, at least in part, to injury of the bile canaliculi. In support of this belief, five patients had cholestasis, which was associated with elevated serum GGT and likely caused by progressing hepatic congestion (Table 2). All of these patients had EOB-MRI grade 4.

To date, unlike patients with chronic viral hepatitis and cirrhosis, no typical laboratory test results have been correlated with the degree of hepatic function in Fontan patients^5^. Among patients with Fontan circulation in one study^30^, 71% had elevated serum GGT levels at a mean of 11 years after the Fontan operation. The elevation was correlated with increased systemic ventricular end-diastolic pressure and pulmonary artery pressure. In addition, in the Schwartz *et al*. study^25^, the GGT level was increased within 3–6 months after the Fontan operation, compared to the preoperative values. A long hospital stay and prolonged use of a chest tube after the Fontan operation were significantly associated with an increased GGT level. Our current findings indicated that an elevation in the serum level of GGT can reveal liver insult in the early stage of FALD. Thus, the serum level of GGT may be a useful biomarker for early-stage FALD.

Several noninvasive modalities are used to assess liver function. Contrast-enhanced computed tomography can delineate a liver with nodules and cirrhosis (e.g. mosaic or reticular patterns and inhomogeneous enhancement^8^), although this modality is insufficient for distinguishing liver tumours and poses the risk of radiation exposure. By contrast, ultrasonography and elastography are radiation-free and low-cost examinations. A drawback is that these modalities do not provide anatomical or histological details of the liver or hepatocyte functions^18^. Magnetic resonance elastography is also accurate for assessing the functional reserve of the liver. However, magnetic resonance elastography requires additional hardware and software, which is an obstacle in its general use^19^.

The EOB-MRI modality provides useful information for the diagnosis and follow up of FALD. A major concern with EOB-MRI is adverse effects of the contrasting agent. A dose of the contrast agent for EOB-MRI is one-half that of the conventional contrast gadolinium MRI; however, anaphylaxis and gadolinium-induced fibrosis (i.e. nephrogenic systemic fibrosis) are risks of the procedure. Therefore, clinicians need to evaluate a patient’s renal function before this examination to avoid renal adverse events.

This study has three limitations. First, histological changes were not directly assessed with liver biopsy. Liver biopsy is not routinely performed for Fontan patients because most patients are administered antiplatelet and/or anticoagulant agents. Evaluating the entire liver by biopsy is difficult, although a comparison of EOB-MRI with liver biopsy would provide further detailed information about the EOB-MRI findings. Second, the sample size of our study was small because the study was limited to young Fontan patients. Third, a hepatic evaluation using EOB-MRI before the Fontan procedure was not conducted. The patients’ liver status before Fontan procedure may have affected the MRI results at the 1-year postoperation follow up.

In conclusion, we elucidated the usefulness of EOB-MRI in patients after the Fontan procedure. This noninvasive technique is potentially a method that could be used to detect early-stage FALD. No reliable biomarker exists that could be used to assess liver function in the Fontan population. Therefore, imaging assessment of the hepatic architecture would be a reliable modality. Further research is warranted to elucidate the full pathophysiological process of FALD, especially longitudinal serial studies to determine the long-term progression of abnormal findings for individual patients.

## Methods

This retrospective study was approved by the Gunma Children’s Medical Center Review Board (Gunma, Japan; Institutional Review Board approval no. 1126). Written informed consent was obtained from the parents or guardians of all patients.

### Patients

We retrospectively reviewed the medical records of 40 consecutive patients who underwent the Fontan procedure between March 2012 and December 2017 in the Gunma Children’s Medical Center (Gunma, Japan). We excluded two patients with a metal implant because they were ineligible for MRI, and we excluded one patient because of extravasation of contrast media during the MRI examination. Hepatic Gd-EOB-DTPA-enhanced MRI was conducted approximately 1 year after the Fontan procedures. All patients underwent a simultaneous follow-up cardiac catheterization and laboratory tests. In addition, 20 patients underwent abdominal ultrasonography with the iE33 ultrasound system (Philips Medical Systems, Andover, MA, USA) around the same time as the MRI.

#### Magnetic resonance protocol

All patients underwent MRI with a 1.5-Tesla scanner (Achieva Nova Dual; Philips, Amsterdam, the Netherlands) with a four-channel torso array coil. With the patient under sedation, all sequences were conducted with respiratory triggering to secure excellent image quality. Two-dimensional fat-suppressed axial T2-weighted turbo spin-echo, diffusion-weighted, single-shot spin-echo images (low b factor, 0 and 8 s/mm^2^) and T1-weighted turbo field echo in-phase images were initially obtained. The contrast agent Gd-EOB-DTPA (i.e. gadoxetic acid; Bayer Schering Pharma, Osaka, Japan) was then intravenously injected at a dose of 25 mol/kg body weight (0.1 mL/kg) and a flow rate of 1 mL/s, followed by a 5-mL saline flush. We used the balanced turbo field-echo sequence to evaluate major veins such as the hepatic and portal veins, and the inferior vena cava, and used the early hepatocyte phase with axial T1-weighted three-dimensional turbo field echo within 5 minutes after the administration of the contrast agent. The parameter of this sequence was as follows: section thickness, 2–2.5 mm; repetition time/echo time (TR/TE), 10/4.6 ms; flip angle, 25°; and number of signals acquired, 2. Approximately 20 minutes after injecting the contrast agent, we obtained the hepatocyte phase by using the same sequence as used in the early hepatocyte phase. In this study, MRI imaging analysis primarily focused on this hepatocyte phase sequence.

### Magnetic resonance imaging analysis

A board-certified paediatric radiologist with expertise in body imaging reviewed the MRI scans. The radiologist was blinded to the patients’ clinical history, except for the patients’ post-Fontan operation status. The MRI scans were evaluated with regard to changes in signal intensity in the hepatobiliary phase of the T1-weighted images. In previous studies^12,31,32^, different patterns of decreased intensity that indicated liver dysfunction were assessed and graded into four levels, based on the extent of enhancement: Grade 1 indicated no decrease in Gd-EOB-DTPA enhancement; Grade 2, a subsegmental decrease; Grade 3, a lobar decrease or a diffuse decrease on four slices or fewer, which indicated a regional decrease; and Grade 4, diffuse poor enhancement on five slices or more. In addition to the contrast abnormality, the MRI images were evaluated for blunt margins in the liver surface, hepatic lobe atrophy or hypertrophy, manifestations of portal hypertension such as oesophageal varix, splenomegaly, and ascites, and delayed excretion of contrast, which indicated cholestasis.

### Measurement of hemodynamic parameters in the cardiac catheterization

The hemodynamic parameters of all patients were assessed by a follow-up cardiac catheterization at 1 year after the Fontan procedure and at the same time as the EOB-MRI. All patients underwent cardiac catheterization with the same anaesthetic protocol, which included the intestinal infusion of midazolam and ketamine and the intravenous infusion of thiopental sodium. In addition, all patients underwent biplane cineventriculography. Their haemodynamic variables were also measured such as CVP, pulmonary artery pressure, PVR, pulmonary artery index^33^, end-diastolic pressure of systemic ventricle, end-diastolic volume index of the systemic ventricle, and cardiac index. Furthermore, the saturation of percutaneous oxygen (SpO_2_) was evaluated at rest. Laboratory testing with venous blood samples obtained by cardiac catheterization consisted of blood counts; blood chemistry such as the levels of AST, ALT, alkaline phosphatase, and GGT; biomarkers of liver function such as hyaluronic acid, type IV collagen, and procollagen III peptide; and brain natriuretic peptide, a biomarker of heart failure.

### Statistical analyses

Continuous variables are expressed as the mean ± the standard deviation or as the median [interquartile range (IQR) 25^th^–75^th^ percentile]. Categorical data are presented as the frequency and percentage. Statistical significance was *P* < 0.05. In addition, the univariate analysis was conducted using the Jonckheere–Terpstra trend test for the continuous variables and the Cochran–Armitage test for the categorical data. Data analysis was performed using commercially available software (SPSS 24.0; SPSS Inc., Chicago, IL).

### Ethical approval

All procedures performed in studies involving human participants were in accordance with the ethical standards of the Gunma Children’s Medical Center Review Board (Gunma, Japan; Institutional Review Board approval no. 1126) and with the 1964 Helsinki declaration and its later amendments or comparable ethical standards.

### Informed consent

Informed consent was obtained from the parents or guardians of all individual participants included in the study.

## Data Availability

No datasets were generated or analysed during the current study.
